# Myelin oligodendrocyte glycoprotein antibody-related autoimmune encephalitis misdiagnosed as acute cerebral infarction: A case report

**DOI:** 10.1097/MD.0000000000049543

**Published:** 2026-07-03

**Authors:** Congling Li, Jianxia Zhi, Yingze Zhu, Yi Zhou

**Affiliations:** a Department of Neurology, Bethune International Peace Hospital, Shijiazhuang, Hebei, People’s Republic of China; b Cancer Hospital Affiliated to Harbin Medical University, Heilongjiang, People’s Republic of China.

**Keywords:** acute cerebral infarction, cortical encephalitis, misdiagnosis, myelin oligodendrocyte glycoprotein antibody-associated disease

## Abstract

**Rationale:**

Myelin oligodendrocyte glycoprotein antibody-associated disease (MOGAD) is an inflammatory demyelinating autoimmune disorder of the central nervous system that exhibits a broad spectrum of known clinical phenotypes, including optic neuritis, myelitis, acute disseminated encephalomyelitis, encephalitis, meningoencephalitis, and brainstem encephalitis. However, new evidence indicates that a small subset of patients may present with cortical cerebral encephalitis (CCE). When CCE manifests as acute unilateral limb weakness, it may closely mimic acute cerebral infarction (ACI), resulting in misdiagnosis and delayed initiation of immunotherapy. Here, we report a case initially diagnosed as ACI and highlight key diagnostic clues and therapeutic considerations that may help to facilitate earlier recognition and appropriate management.

**Patient concerns:**

A young woman was admitted with left-sided limb weakness and was initially diagnosed with ACI.

**Diagnoses:**

The final diagnosis was myelin oligodendrocyte glycoprotein (MOG) antibody-related autoimmune encephalitis, which was established by integrating the clinical presentation, contrast-enhanced brain magnetic resonance imaging (MRI) findings, cerebrospinal fluid (CSF) antibody testing, and systematic exclusion of alternative etiologies.

**Interventions:**

After an initial misdiagnosis, the patient received neuroprotective agents without meaningful improvement. Subsequent lumbar puncture and CSF testing supported the diagnosis of MOG antibody-related autoimmune encephalitis. Treatment was then adjusted to include both high-dose intravenous corticosteroid pulse therapy and immunotherapy, which resulted in marked clinical improvement.

**Outcomes:**

The patient’s condition remained stable at 2-month follow-up, with no disease progression. Contrast-enhanced MRI showed no abnormalities in the unilateral cerebral cortex.

**Lessons:**

CCE associated with MOGAD may be misdiagnosed as ACI, particularly when focal motor deficits are the predominant presenting feature. Early recognition, prompt diagnostic evaluation, and timely initiation of immunotherapy are crucial for improving clinical outcomes. Greater clinician awareness and improved neuroimaging-based differentiation between ACI and CCE are therefore needed. In patients with stroke-mimicking presentations but atypical imaging findings, contrast-enhanced MRI and cerebrospinal fluid testing for MOG immunoglobulin G should be prioritized. High-dose steroid pulse therapy combined with immunotherapy remains the recommended first-line treatment for suspected MOG antibody-related autoimmune encephalitis.

## 1. Introduction

In recent years, myelin oligodendrocyte glycoprotein (MOG) antibody-associated disease (MOGAD) has been increasingly recognized as a distinct clinical entity,^[1]^ and MOG antibody-related autoimmune encephalitis, in particular, is one of its phenotypes that is more commonly reported in adolescents and young adults, especially females.^[2]^ Typical clinical manifestations include seizures, headache, fever, and cortical symptoms corresponding to lesion location.^[3]^ On magnetic resonance imaging (MRI), a characteristic finding on T2-fluid-attenuated inversion recovery (T2-FLAIR) sequences is gyriform cortical hyperintensity during the acute phase, and approximately 30% of patients show leptomeningeal enhancement in adjacent sulci.^[4]^ Unilateral cortical encephalitis with seizures is now recognized as a distinct phenotype within MOGAD as well.^[5]^ However, published reports of cortical cerebral encephalitis (CCE) remain limited, and early misdiagnosis is common. This article reports a case of CCE that was misdiagnosed as cerebral infarction at the initial presentation.

## 2. Case presentation

**Figure 1.**
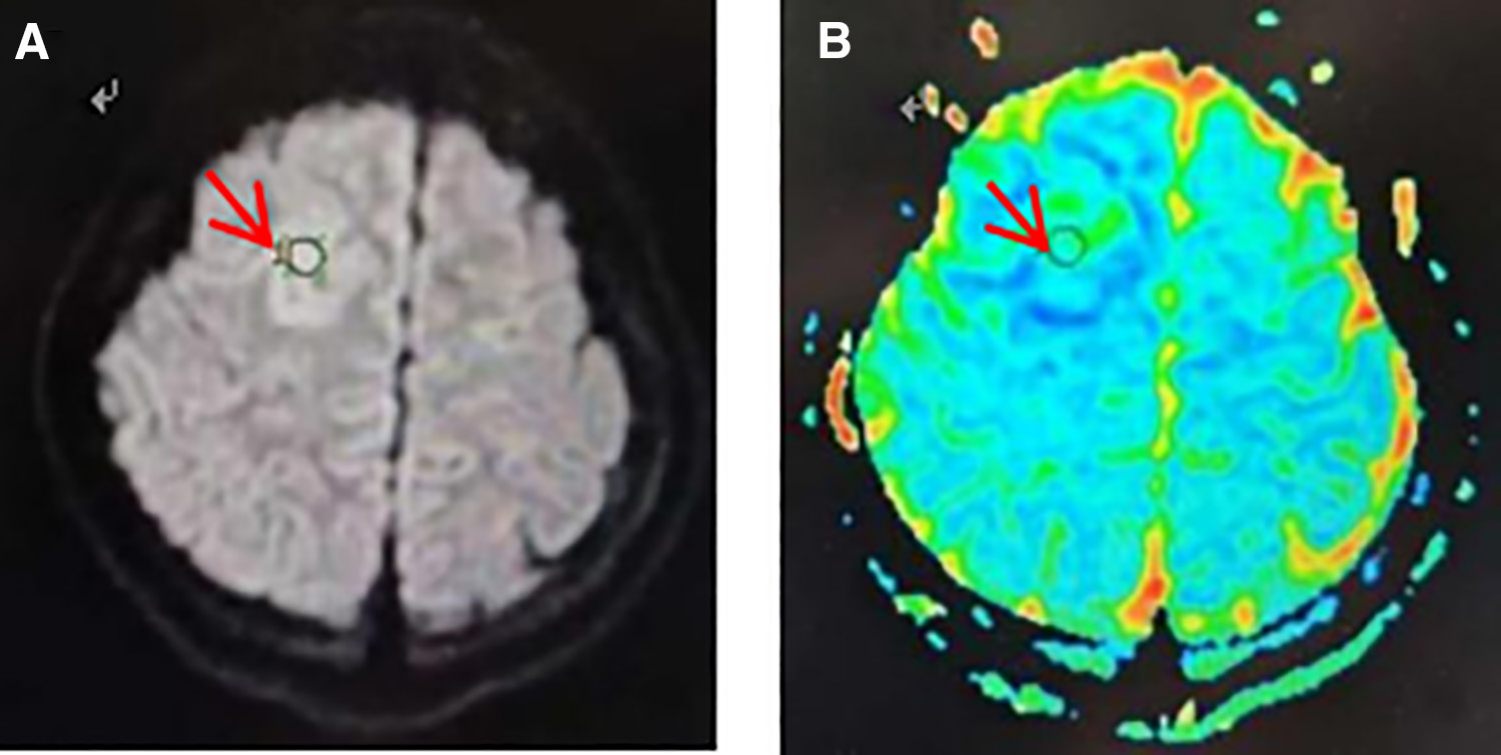
(A) The transverse sequence of the brain DWI; (B) The ADC perfusion sequence. ADC = apparent diffusion coefficient, DWI = diffusion-weighted imaging, MRI = magnetic resonance imaging.

**Figure 2.**
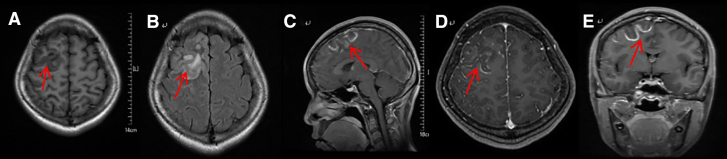
(A) The T1WI sequence of the transverse cranial section; (B) The T2WI sequence of the transverse craniocerebral section; (C) T1WI sequence of the cranial magnetic resonance imaging enhances the sagittal position; (D) Enhanced transverse position of T1WI sequence in cranial magnetic resonance imaging; (E) T1WI sequence of the cranial MRI enhances the coronal position. MRI = magnetic resonance imaging, T1WI = T1-weighted imaging, T2WI = T2-weighted imaging.

**Figure 3.**
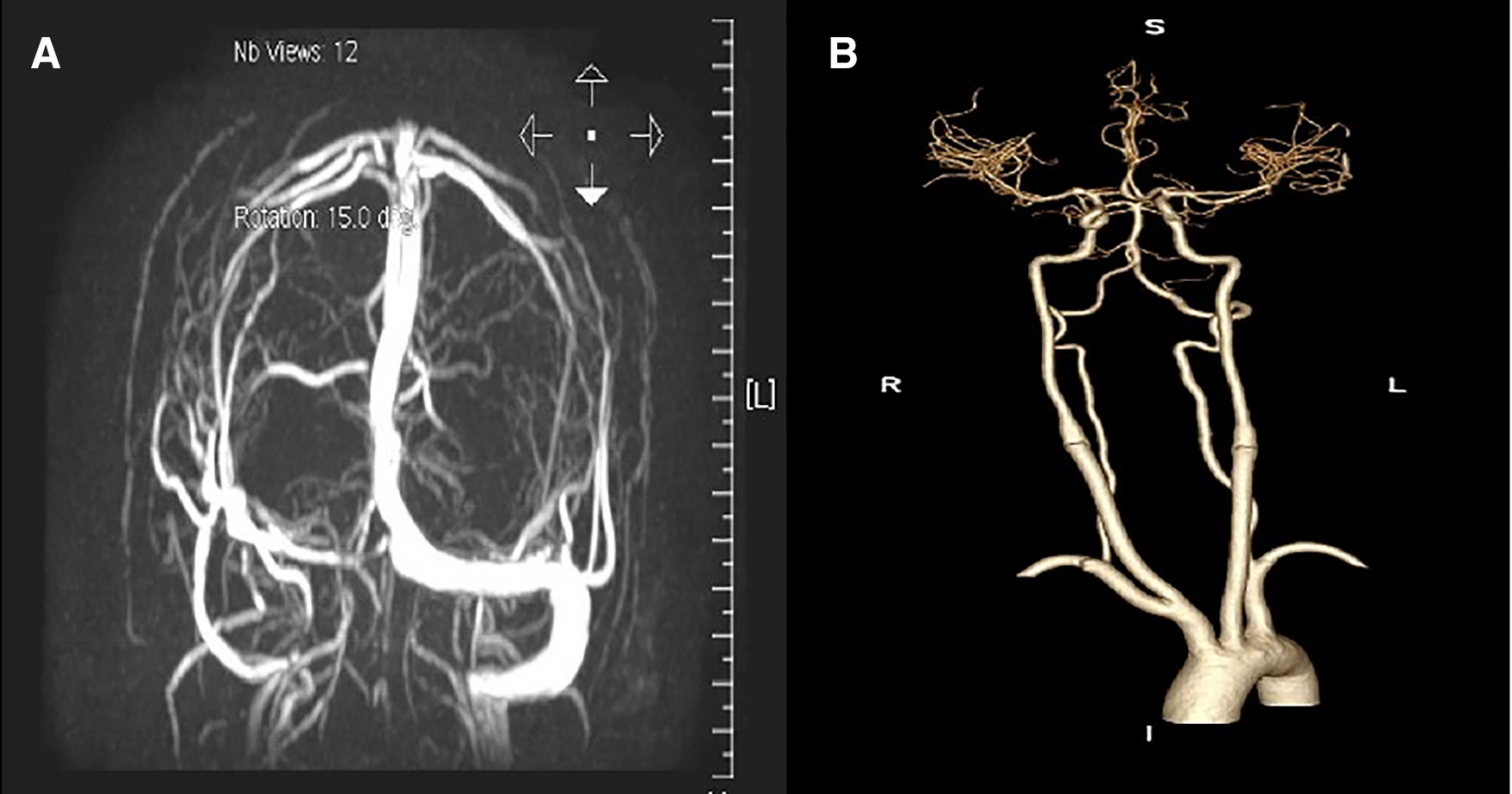
(A) Cranial magnetic resonance venography: The right transverse sinus and sigmoid sinus are relatively slender, suggesting left-sided dominance; (B) Computed tomography angiography of the head and neck: The cerebral vessels showed no significant stenosis.

**Figure 4.**
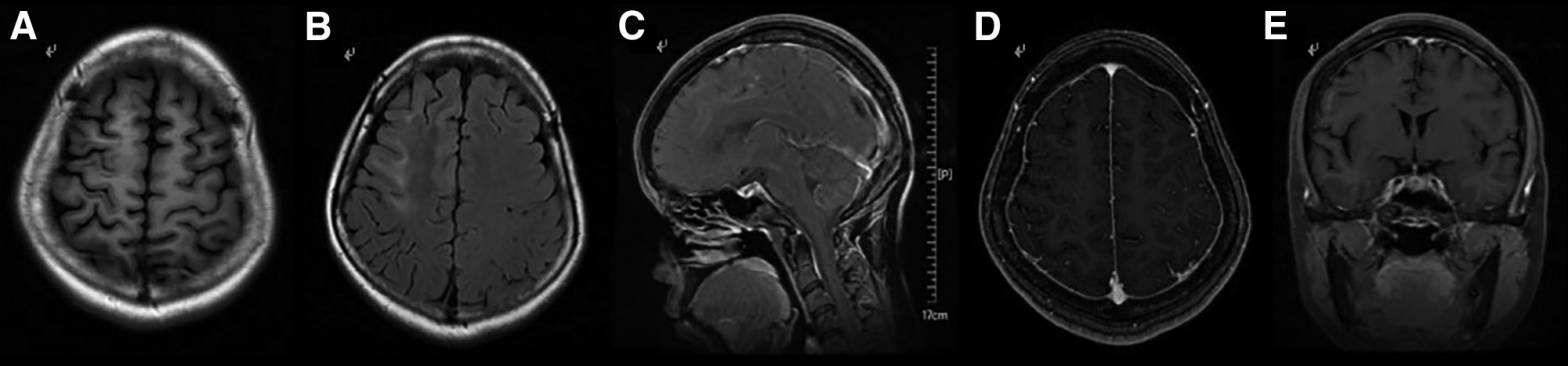
(A) The T1WI sequence of the cranial transverse section; (B) The T2WI sequence of the transverse craniocerebral section; (C) The T1WI sequence enhancement of the sagittal position; (D) Transverse enhancement of the T1WI sequence in cranial magnetic resonance imaging; (E) Coronal enhancement of the T1WI sequence in cranial magnetic resonance imaging. T1WI = T1-weighted imaging, T2WI = T2-weighted imaging.

**Figure 5.**
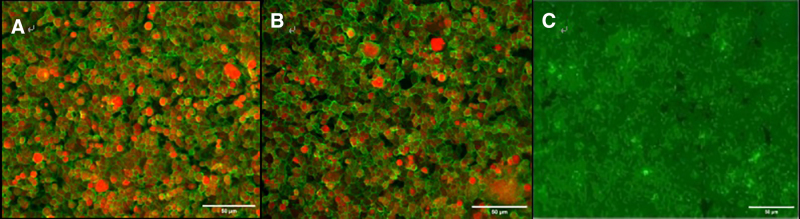
(A) Dual-color fluorescence overlay map of the patient’s first serum tests (MOG-IgG: 1:100. Scale bars: 50 μm. Methodology: Live cell-based assay); (B) Dual-color fluorescence overlay map of the first cerebrospinal fluid tests (MOG-IgG: 1:32. scale bars: 50 μm. Methodology: Live cell-based assay); (C) The second cerebrospinal fluid test 2 mo later (MOG-IgG: negative; scale bars: 50 μm; methodology: Transfected cell-based assay). Titer determination method: The initial dilution for serum was 1:10 (positive ≥ 1:10); the initial dilution for the CSF was 1:1 (positive ≥ 1:1). The samples were tested in different assay runs; this seroconversion is reported qualitatively rather than as a quantitative titer change.

**Figure 6.**
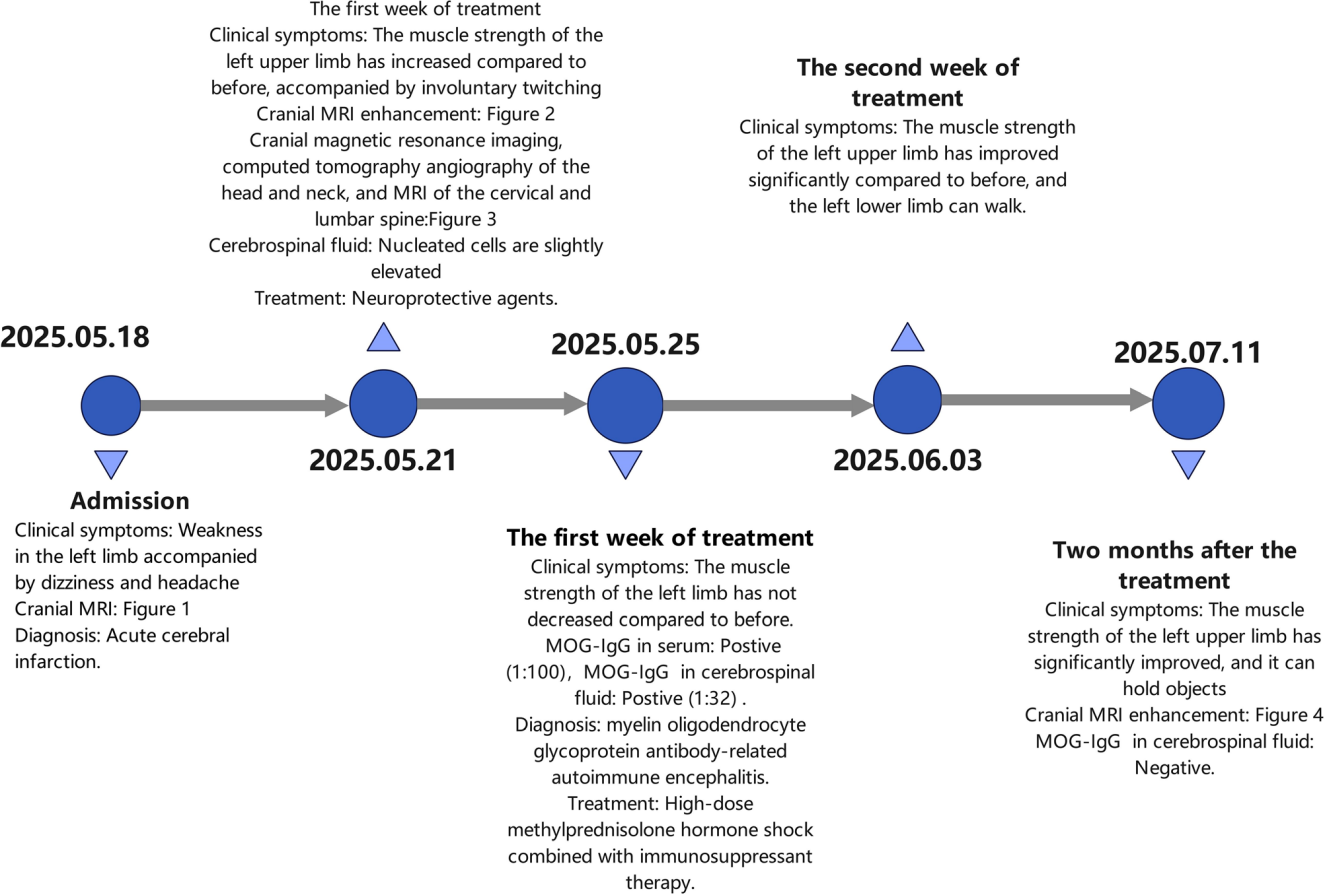
The diagnostic and therapeutic timeline.

A 30-year-old woman presented to Bethune International Peace Hospital emergency department on May 18, 2025, with progressive left-sided weakness over the past week that had worsened over the preceding day. The weakness developed without an obvious trigger and was associated with gait instability, difficulty lifting objects, dizziness, and headache. She denied syncope, impaired consciousness, seizures, nausea, vomiting, transient visual loss, vertigo, dysarthria, choking while drinking, and dysphagia. Her symptoms fluctuated intermittently but showed overall progression. Initial brain MRI demonstrated a right frontal lobe lesion with diffusion-weighted imaging (DWI) hyperintensity, which was interpreted as acute cerebral infarction (ACI) (Fig. 1). She was treated empirically with butylphthalide sodium chloride and edaravone dexborneol but showed minimal clinical improvement and was thus subsequently admitted to the Department of Neurology for further evaluation with a preliminary diagnosis of cerebral infarction. Her past medical history was notable only for excision of a pigmented nevus on the plantar surface of the right foot on April 30, 2025. Personal and family histories were otherwise unremarkable. Upon neurological examination, muscle strength in the left upper and lower extremities was Medical Research Council grade 4, with ipsilateral positive Babinski and Chaddock signs. Left-sided dysmetria was present on finger-to-nose and heel-to-shin testing. Routine laboratory tests, including complete blood count, liver and renal function tests, and infectious disease screening, were normal. Electrocardiography and chest radiography showed no significant abnormalities. Repeat contrast-enhanced brain MRI on May 21, 2025 showed abnormal gyral signal in the right frontal lobe, with mild hypointensity on T1-weighted imaging and hyperintensity on T2-weighted and T2-FLAIR sequences that were accompanied by linear gyriform enhancement after contrast administration (Fig. 2). Cranial magnetic resonance venography, computed tomography angiography of the head and neck (Fig. 3), and MRI of the cervical and lumbar spine were unremarkable. Lumbar puncture revealed clear cerebrospinal fluid (CSF) with an opening pressure of 80 mm H_2_O; CSF analysis showed lymphocytic pleocytosis 94 cells/µL; 91% lymphocytes; and Pandy test and CSF glucose, protein, and chloride levels were normal. Extensive pathogen testing was negative. After 1 week of treatment, there was still no significant improvement in left-sided muscle strength. Autoimmune encephalitis antibody panels and oligoclonal bands in serum and CSF were obtained on May 25, 2025 and both were negative. However, MOG antibodies were positive in both serum and CSF. The serum MOG antibody titer was 1:100 as determined by a cell-based transfection assay, and the CSF MOG antibody titer was 1:32 (Fig. 5A and B) (Table 1). These findings supported revision of the diagnosis to MOG antibody-related autoimmune encephalitis. The patient was then treated with intravenous methylprednisolone sodium succinate in combination with intravenous immunoglobulin. During hospitalization, left-sided muscle strength remained stable without further deterioration, and the patient was discharged on a tapering regimen of oral methylprednisolone for maintenance (Table 2). A residual abnormal signal was noted along the right frontal gyrus during a follow-up brain MRI with and without contrast performed on July 11, 2025, which appeared mildly hypointense on T1-weighted images and hyperintense on T2-weighted and T2-FLAIR sequences (Fig. 4), without gadolinium enhancement. Repeat lumbar puncture showed normalization of cerebrospinal fluid parameters, and myelin oligodendrocyte glycoprotein immunoglobulin G (MOG-IgG) became negative in the CSF (Fig. 5C). Clinically, left-limb strength improved substantially. The diagnostic and therapeutic timeline is summarized in Figure 6.

**Table 1:** Serum and cerebrospinal fluid autoimmune antibody panel results.

Panel of autoimmune encephalitis antibodies			
Antibody	Serum results	CSF results	Testing method
NMDAR-IgG	Negative	Negative	Transfected cell-based assay
AMPAR1-IgG	Negative	Negative	Transfected cell-based assay
AMPAR2-IgG	Negative	Negative	Transfected cell-based assay
LGI1-IgG	Negative	Negative	Transfected cell-based assay
CASPR2-IgG	Negative	Negative	Transfected cell-based assay
GABABR-IgG	Negative	Negative	Transfected cell-based assay
DPPX-IgG	Negative	Negative	Transfected cell-based assay
IgLON5-IgG	Negative	Negative	Transfected cell-based assay
GlyR1-IgG	Negative	Negative	Transfected cell-based assay
D2R-IgG	Negative	Negative	Transfected cell-based assay
GAD65-IgG	Negative	Negative	Transfected cell-based assay
mGluR5-IgG	Negative	Negative	Transfected cell-based assay
mGluR1-IgG	Negative	Negative	Transfected cell-based assay
GABAARα1-IgG	Negative	Negative	Transfected cell-based assay
GABAARβ3-IgG	Negative	Negative	Transfected cell-based assay
Neurexin3α-IgG	Negative	Negative	Transfected cell-based assay
Oligoclonal band			
AQP4-IgG	Negative	Negative	Transfected cell-based assay
**MOG-IgG**	**Positive (1: 100**)	**Positive (1: 32**)	**Live cell-based assay**
MBP-IgG	Negative	Negative	Transfected cell-based assay
GFAP-IgG	Negative	Negative	Transfected cell-based assay
AQP1-IgG	Negative	Negative	Transfected cell-based assay

AMPAR1-IgG = α-amino-3-hydroxy-5-methyl-4-isoxazolepropionic acid receptor 1 immunoglobulin G, AMPAR2-IgG = AMPAR2-IgGα-amino-3-hydroxy-5-methyl-4-isoxazolepropionic acid receptor 2 immunoglobulin G, AQP1-IgG = aquaporin-1 immunoglobulin G, AQP4-IgG = aquaporin‑4 immunoglobulin G, CASPR2-IgG = contactin-associated protein-like 2 immunoglobulin G, CSF = cerebrospinal fluid, D2R-IgG = dopamine D2 receptor immunoglobulin G, DPPX-IgG = dipeptidyl-peptidase-like protein 6 immunoglobulin G, GABAARα1-IgG = gamma-aminobutyric acid A receptor alpha 1 subunit immunoglobulin G, GABAARβ3-IgG = gamma-aminobutyric acid A receptor beta 3 subunit immunoglobulin G, GABABR-IgG = gamma-aminobutyric acid B receptor immunoglobulin G, GAD65-IgG = glutamic acid decarboxylase 65 immunoglobulin G, GFAP-IgG = glial fibrillary acidic protein immunoglobulin G, GlyR1-IgG = glycine receptor alpha 1 subunit immunoglobulin G, IgLON5-IgG = IgLON family member 5 immunoglobulin G, LGI1-IgG = leucine-rich glioma-inactivated 1 immunoglobulin G, MBP-IgG = myelin basic protein immunoglobulin G, mGluR1-IgG = metabotropic glutamate receptor 1 immunoglobulin G, mGluR5-IgG = metabotropic glutamate receptor 5 immunoglobulin G, MOG-IgG = myelin oligodendrocyte glycoprotein immunoglobulin G, Neurexin3α-IgG = neurexin-3-alpha immunoglobulin G, NMDAR-IgG = N-methyl-D-aspartate receptor immunoglobulin G.

**Table 2:** Summary of therapeutic interventions.

Drug	Dose	Route of administration	Frequency	Duration
Methylprednisolone sodium succinate for injection	1000 mg	Intravenous infusion	Once daily	3 d
	480 mg	Intravenous infusion	Once daily	5 d
	240 mg	Intravenous infusion	Once daily	5 d
	120 mg	Intravenous infusion	Once daily	3 d
Methylprednisolone tablets	60 mg	Oral	Once daily	3 mo
Intravenous human immunoglobulin	22.5 g	Intravenous infusion	Once daily	5 d

## 3. Discussion

López-Chiriboga et al proposed the concept of MOGAD in 2018 when they reported that approximately 6.7% of affected patients may present with encephalitis-like manifestations.^[2]^ MOG antibody-related autoimmune encephalitis is, therefore, considered a rare clinical phenotype within the MOGAD spectrum. MOG-IgG is predominantly related to the IgG1 subtype immunoglobulin and has strong complement-activating capacity, suggesting a potential role in antibody-mediated demyelination.^[6,7]^ Mounting evidence suggests that environmental factors such as infection, vaccination, and tissue injury may act as triggers for disease onset or relapse.^[8]^ In this context, surgical procedures may promote the release of sequestered antigens or induce immune activation, thereby facilitating autoimmune responses. In the present case, neurological symptoms occurred 18 days after excision of a pigmented nevus. Although a causal relationship cannot be definitively established, the temporal association raises the possibility that surgical stress or tissue injury contributed to disease onset.

The patient presented with acute left-sided limb weakness without fever, seizures, or clear disturbances of consciousness, which indicated that encephalopathic features were not prominent. This clinical presentation differs significantly from the classical phenotype of MOG antibody-related cortical encephalitis or FLAIR-hyperintense lesions in cortical encephalitis with MOG antibodies (FLAMES).^[9]^ FLAMES is a clinico-radiological syndrome of MOGAD first described by Ogawa in 2017^[10]^ whose core features include acute or subacute onset of cortical encephalitis symptoms, typically including seizures, especially focal motor seizures or status epilepticus, fever, headache, and encephalopathic manifestations such as confusion and cognitive decline. Brain MRI typically shows unilateral or bilateral cortical hyperintensity on T2-FLAIR, which may be accompanied by leptomeningeal or cortical enhancement, without involvement of the deep white matter.^[4]^ This nomenclature has since been adopted by many subsequent studies and incorporated into the diagnostic framework of MOGAD. However, our patient had neither seizures nor fever; she presented only with acute left-sided limb weakness and ataxia. Later evaluation revealed slowed mentation, but overall she lacked the classical “cortical encephalitis triad” of FLAMES. Therefore, her case does not conform to the classic FLAMES phenotype but rather represents a MOGAD variant with a stroke-like focal motor deficit as the main manifestation.

This stroke-mimicking presentation without seizures, fever, or prominent encephalopathy is rare in MOGAD but carries significant differential diagnostic importance. Clinically, this presentation can represent one of 2 possibilities. First, there may exist a clinical subtype within the MOGAD spectrum that differs from typical FLAMES, characterized by a pure motor deficit or pseudostroke as the prominent feature, that lacks seizures due to increased cortical excitability and systemic inflammatory response seen in typical FLAMES. Such patients may be more readily misdiagnosed with ACI, especially in young individuals. Second, this presentation could merely represent a diagnostic pitfall within the same disease spectrum in which the patient may have subtle or subclinical encephalopathic or seizure manifestations such as slowed mentation, unnoticed focal cognitive changes, or a lesion in the prefrontal or motor cortex that causes motor symptoms to overshadow other cortical symptoms. In the present case, although the patient did not have seizures, she was ultimately diagnosed with MOG antibody-related autoimmune encephalitis. Imaging showed right frontal cortical-subcortical lesions with linear gyriform enhancement, CSF showed lymphocytic inflammation, and she responded well to glucocorticoid therapy. This clinical course suggests that the stroke-like presentation of MOGAD may not represent an independent subtype. Rather, it more likely reflects silent cortical encephalitis in which the pathology is confined to or predominantly involves motor-associated cortical regions, without widespread activation of the limbic system or spread to epileptic networks.^[11]^

From a pathophysiological perspective, the stroke-mimicking mechanism may be related to localized disruption of the blood-brain barrier and MOG antibody-mediated astrocyte or oligodendrocyte injury confined to the motor cortex, without direct interference with neuronal excitability circuits or systemic release of inflammatory cytokines.^[12]^ Of note, a few previously reported MOGAD cases have presented with isolated hemiparesis or monoparesis without seizures or fever, indicating that although this stroke-mimicking phenotype is atypical, it does exist within the MOGAD spectrum.^[13]^ Therefore, we propose that the presentation of this case does not constitute an independent clinical subtype, but rather an easily overlooked diagnostic pitfall within the MOGAD spectrum. Specifically, when the epileptic and febrile features of cortical encephalitis are absent, a focal motor deficit can easily be mistaken for ischemic stroke, particularly when DWI hyperintensity is present.

A diagnosis of MOG antibody-related autoimmune encephalitis requires the integration of a core clinical syndrome, serological evidence of MOG-IgG positivity, supportive neuroimaging features, and reasonable exclusion of alternative diagnoses.^[1]^ Our case met these criteria through a structured diagnostic work-up. Clinically, the patient presented with an acute encephalopathic syndrome accompanied by focal neurological deficits in the form of left-limb weakness and ataxia, which is a recognized core phenotype of MOGAD. Serologically, serum MOG-IgG was detected at a titer of 1:100, thus providing key supportive evidence, and MOG-IgG was also positive in the CSF at a titer of 1:32, suggesting central nervous system-directed humoral activity. Neuroimaging further supported the diagnosis. Brain MRI demonstrated right frontal cortical-subcortical lesions with linear gyriform enhancement, a characteristic pattern of the encephalitic phenotype of MOGAD, but it lacked features typical of multiple sclerosis such as Dawson’s finger-like lesions. MR venography and computed tomography angiography were normal, providing evidence against venous thrombosis or major vascular pathology. Furthermore, CSF analysis showed lymphocytic pleocytosis consistent with immune-mediated inflammation; other autoimmune encephalitides were systematically excluded. Although the initial DWI hyperintensity raised concern for infarction, the subsequent evolution did not support a purely vascular event and was more consistent with MOGAD-associated cortical perivascular inflammation. The patient showed a favorable response to high-dose corticosteroid therapy, with improvement in muscle strength, resolution of contrast enhancement, and seroconversion to MOG-IgG negativity in the CSF.

The main diagnostic challenge in this case lay in the stroke-mimicking mechanism. Acute ischemic infarction typically conforms to a vascular territory distribution and shows high signal intensity on DWI with a marked reduction in apparent diffusion coefficient values. Early contrast enhancement is uncommon, and the lesion evolution is relatively stereotyped. In contrast, MOG antibody-related encephalitis often involves the cortex, subcortical white matter, deep white matter, basal ganglia, thalamus, and brainstem. Lesions are frequently ill-defined, and apparent diffusion coefficient values within DWI-hyperintense regions can be heterogeneous. Enhancement patterns are also variable and may include leptomeningeal, linear, nodular, or ring-like enhancement.^[3,8]^ Research has shown that acute ischemic infarction is typically associated with hypoperfusion, whereas encephalitic lesions more often demonstrate iso-to hyperperfusion with a heterogeneous pattern in arterial spin labeling (ASL).^[14]^ These differences provide important evidence for differential diagnosis. Clinicians must consider that encephalitis can mimic acute ischemic stroke in young patients without clear cerebrovascular risk factors who present with stroke-like symptoms, particularly when the clinical course is atypical or when DWI hyperintensity is observed. In such circumstances, actively performing brain MRI with ASL is critical for differentiating stroke mimics.

Unfortunately in this case, we did not perform an MRI ASL sequence, which provides a lesson for future diagnosis and treatment. Of note, however, is that unilateral cortical encephalitis is not specific to MOGAD. Viral encephalitis, Rasmussen encephalitis, CJD, and other autoimmune encephalitides can present with a similar unilateral cortical pattern. Rasmussen encephalitis is characterized by refractory epilepsy and transient hemiparesis, with MRI showing progressive unilateral hemispheric atrophy.^[15]^ NMDAR antibody-related encephalitis frequently affects young women, with an ovarian teratoma identified in approximately 40–60% of cases. In such cases, a prodromal phase with headache and fever may occur, and psychiatric and behavioral disturbances are often the earliest manifestations. Furthermore, cases of anti-NMDAR and anti-MOG antibody overlapping encephalitis have also been reported.^[16]^

Management of MOGAD consists of acute-phase therapy and remission-phase maintenance treatment. Most patients respond favorably to glucocorticoids, with retrospective cohort studies reporting clinical response rates that exceed 50%.^[17,18]^ For severe acute attacks, intravenous immunoglobulin or early initiation of plasma exchange may be added.^[19]^ Therefore, early recognition of encephalitis in young and middle-aged adults is crucial.

This report has several limitations. First, as a single case, the observations may not be generalizable to all patients with MOGAD who present with stroke-like symptoms. Second, the follow-up duration of approximately 2 months is relatively short. However, we confirmed that the patient is enrolled in an ongoing longitudinal follow-up program at our institution, and longer-term data will be reported separately in a future publication. Third, this study is limited by the lack of electroencephalography data. Future prospective studies should incorporate continuous electroencephalography monitoring in order to characterize the electrophysiological correlates of this phenotype. Despite these limitations, this case highlights an important diagnostic pitfall and underscores the need for heightened awareness of MOGAD in stroke-mimicking presentations.

## Acknowledgments

The authors thank AiMi Academic Services (www.aimieditor.com) for English language editing and review services.

## Author contributions

**Conceptualization:** Yi Zhou.

**Data curation:** Yingze Zhu, Yi Zhou.

**Formal analysis:** Yingze Zhu.

**Investigation:** Jianxia Zhi.

**Methodology:** Congling Li.

**Resources:** Jianxia Zhi.

**Supervision:** Yi Zhou.

**Visualization:** Congling Li.

**Writing – original draft:** Congling Li.

**Writing – review & editing:** Yi Zhou.
